# Impact of palliative care integration on end-of-life resource utilization and costs in oncology patients: a single-center analysis in Jordan

**DOI:** 10.3389/fpubh.2026.1801343

**Published:** 2026-04-28

**Authors:** Ghadeer Alarjeh, Waleed Alrjoub, Mousa Abdal-Rahman, Abdulrahman Shamieh, Mohammed O. Al-Bssol, Asem Mansour, Omar Shamieh

**Affiliations:** 1 Department of Palliative Care, King Hussein Cancer Center, Amman, Jordan; 2 Center for Palliative and Cancer Care in Conflict (CPCCC), King Hussein Cancer Centre, Amman, Jordan; 3 School of Medicine, University of Jordan, Amman, Jordan; 4 Office of Scientific Affairs and Research, King Hussein Cancer Centre, Amman, Jordan; 5 Office of Director General, King Hussein Cancer Center, Amman, Jordan

**Keywords:** cost analysis, end-of-life, healthcare utilization, oncology, palliative care, palliative referral

## Abstract

**Introduction:**

Evidence from Middle Eastern cancer centers on the impact of palliative care on end-of-life practices remains limited. This study evaluated healthcare utilization, diagnostic costs, and timing of palliative care referral requests at a tertiary cancer center in Jordan.

**Materials and methods:**

We conducted a retrospective cohort study including all 299 adult cancer patients who died between February 2017 and May 2018. Patients were categorized based on the clinical service responsible at death (oncology vs. palliative care). Demographic and clinical characteristics, reasons for final hospitalization, and costs of laboratory and radiologic exams performed in the last seven days of life were extracted. Descriptive and univariate analyses were performed.

**Results:**

Of 299 patients, 151 (50.5%) were managed by palliative care at death. The mean (SD) age was 57.1 ± 14.3 years. The most common reasons for final admission were decreased level of consciousness 60 (20.1%) and pain crisis 57 (19.1%). Among the 148 oncology patients, 43 (29.1%) had documented palliative care referral requests but remained under oncology care at the time death. In the last seven days of life, 1,623 diagnostic exams were performed at a total cost of 186,572 USD. Oncology-managed patients had higher diagnostic intensity and costs compared to palliative patients (mean cost 856.58 vs. 397.38 USD; mean exams 6.6 vs. 4.2; both *p* < 0.001). Exams were performed closer to death in oncology patients (mean 0.39 vs. 2.92 days; *p* < 0.001). Referral requests were associated with longer referral-to-death intervals (median 14 vs. 1 day; *p* < 0.001).

**Discussion:**

Palliative care involvement was associated with lower diagnostic intensity and costs at end of life. However, referral requests were often late or not translated into active palliative management. Earlier and more consistent integration of palliative care may improve resource utilization and align care with patient-centered goals.

## 1 Introduction

Worldwide, end-of-life (EOL) care represents a critical aspect of oncology and palliative care medicine. When patients approach death, balancing aggressive interventions with comfort-oriented care remains challenging for clinicians (1). Earle et al. identified frequent hospital admissions, aggressive treatments such as chemotherapy, and late or absent palliative referral near death as indicator of poor quality of life care (2). Palliative care studies have shown that excessive laboratory and imaging exams near death often contribute little to clinical benefit while increasing patient distress and healthcare costs (3, 4). Early palliative integration can mitigate these issues by aligning care with patient goals and improving outcomes (5).

Cancer care costs have risen sharply over recent decades, creating a substantial financial burden on health systems worldwide. In the United States, the average cost of cancer care per patient ranges from USD 150,000 to more than USD 300,000, depending on cancer type and treatment intensity, with national expenditures projected to exceed USD 246 billion by 2030 (6). In the Middle East, where many healthcare systems operate under constrained budgets, the escalating costs of diagnostics, targeted therapies, and supportive care pose significant challenges to the sustainability of cancer care. Limited financial resources, combined with a rising cancer incidence, have intensified pressure on already overextended systems and heightened concerns regarding long-term healthcare viability. The economic burden is substantial; projections estimate that without accelerated investment in prevention and treatment, the cost of women's cancers alone in the region could reach approximately US $408 billion by 2050 (7).

Treating physicians play a central role in determining the cost of exams and whether it is beneficial or not for patients at the end of life (8). However many studies found that unnecessary high cost images done frequently for patients at EOL (9). Kwok et al. (10) reported that approximately one-quarter of patients with stage IV cancer undergo procedures during the final month of life, reflecting a high intensity of care near death. Similarly, a large matched-cohort study of over 376,000 decedent's demonstrated steadily increasing laboratory use as death approached, culminating in a sharp escalation during the final month, whereas utilization among non-decedents remained stable (11). Despite this growing international evidence, data from Middle Eastern cancer centers remain scarce. In Jordan, Abdel-Razeq et al. (12) found that 18.1% of cancer patients at a tertiary cancer center received chemotherapy in the last 30 days of life and 8.3% in the last 14 days, with younger individuals and those with hematologic malignancies more likely to receive aggressive treatment, while palliative referral was associated with significantly lower end-of-life chemotherapy use. Building on these gaps, the present study evaluates near-death care at King Hussein Cancer Center (KHCC) by comparing oncology and palliative cohorts in terms of healthcare utilization, associated costs, and timing of palliative referral. This study aimed to compare end-of-life diagnostic utilization, associated costs, and timing of palliative referral between oncology-managed and palliative care patients at KHCC.

## 2 Materials and methods

### 2.1 Design and setting

This retrospective observational study was conducted at KHCC, a leading tertiary oncology institution that provides care for approximately 60% of cancer patients in Jordan. Located in the capital city of Amman, KHCC delivers comprehensive cancer services for both adult and pediatric populations, serving patients from across Jordan, neighboring countries, and refugee communities. As a non-profit institution, KHCC offers fully government-covered treatment for Jordanian patients, ensuring equitable access to specialized oncology and supportive care (13).

### 2.2 Data source

Data on the frequency and cost of laboratory and radiologic exams performed during the final 7 days of life were obtained from multiple institutional sources, including the KHCC Patient Journey and Health Information database, electronic medical records, and the finance department. Extracted variables encompassed patient demographics (age and sex), cancer type, disease stage, diagnostic utilization patterns, associated costs, and the interval between palliative referral and death. For the purposes of this study, palliative referral was defined by the date on which a referral request was initiated, rather than the date of formal acceptance or transition to palliative care as the primary managing service. Ethical approval for this study was obtained from the Institutional Review Board of King Hussein Cancer Center (Reference: 18 KHCC 29).

### 2.3 Study population selection

We included all adult cancer patients who died in the hospital over a 15-month period from February 2017 to May 2018, regardless of their gender, cancer type, stage, or diagnosis. All patients had been admitted prior to death and were classified into oncology or palliative care (hospice) cohorts according to the service managing their care at the time of death. Admissions were not random. Patients were generally admitted to oncology for disease-directed management, while those already under palliative care prior to admission continued under that service. Some oncology patients were later referred and transferred to palliative care if their symptom burden or end-of-life needs increased, whereas others remained under oncology care until death.

### 2.4 Analysis

Continuous variables were summarized using means ± standard deviations (SD) or medians with interquartile ranges, as appropriate, to describe central tendency and variability. Categorical variables were reported as frequencies and percentages. Differences between oncology and palliative cohorts were assessed using independent-samples *t*-tests for normally distributed continuous variables and Mann–Whitney U tests for non-normally distributed variables. Associations between categorical variables were evaluated using chi-square or Fisher's exact tests, as appropriate. Statistical significance was defined as a two-sided *p*-value < 0.05. All statistical analyses were conducted using SAS software version 9.4 (14). Graphical analyses and data visualizations were generated using RStudio (15), ensuring reproducibility and alignment with contemporary analytical standards in clinical research.

## 3 Results

### 3.1 Patients characteristics

The data of 299 adult cancer patients were included, of whom 151 (50.5%) died under palliative care service, 150 (50.2%) were male, with a mean age at death of 57.13 ± 14.25 years. Stage IV disease was present in 284 patients (94.6%) at the final admission. The leading indications for the final hospitalization were decreased level of consciousness 60 (20.1%) and pain crisis 57 (19.1%). Gastrointestinal cancers were most frequent 87 (29.1%), followed by breast cancer 54 (18.1%). Palliative care referral was requested for 194 patients (64.9%), yet 43 oncology patients (29.1% of the oncology cohort) were never actually referred to palliative care prior to death as shown in Table 1.

**Table 1:** Patients characteristics (*N* = 299).

Variable	Frequency (%)
Gender	
Female	149 (49.8%)
Male	150 (50.2%)
Age at death (Group)	
Above or equal 57.9	150 (50.2%)
Less than 57.9	149 (49.8%)
Mean (SD)	57.13(14.3)
Cancer type	
Gastrointestinal	87 (29.1%)
Breast cancer	54 (18.1%)
Genitourinary	46 (15.4%)
Lung cancer	35 (11.7%)
Central nervous system	20 (6.7%)
Gynecological	14 (4.7%)
Head & neck	11 (3.7%)
Hematological	10 (3.3%)
Sarcoma	15 (5%)
Others	7 (2.3%)
Stage at diagnosis	
I	46 (15.4%)
II	43 (14.4%)
III	64 (21.4%)
IV	146 (48.8%)
Last admission stage	
I	4 (1.3%)
II	1 (0.3%)
III	11 (3.7%)
IV	283 (94.6%)
Reason for admission	
Decrease level of consciousness	60 (20.1%)
Pain	57 (19.1%)
Shortness of breath	39 (13.1%)
Fever	20 (6.7%)
Decrease oral intake	12 (4.0%)
Bleeding	11 (3.7%)
Procedure	11 (3.7%)
General weakness	15 (5.1%)
Electrolyte imbalances	10 (3.3%)
Seizure	8 (2.7%)
Other	56 (18.7%)
Died	
Oncology	148 (49.5%)
Palliative	151 (50.5%)
Palliative Referral	
No	105 (35.1%)
Yes	194 (64.9%)

### 3.2 Comparison of baseline characteristics between oncology and palliative care

Patients' characteristics are presented in Table 2. There were no statistically significant differences between the groups in age at death (*p* = 0.452), gender (*p* = 0.118), last admission stage (*p* = 0.434), or reasons for admission (all *p* > 0.05). The majority of patients in both groups had advanced disease at the time of last admission, with stage IV observed in 94.6% of oncology patients and 94.7% of palliative patients. However, significant differences were noted in cancer type (*p* = 0.031) and stage at diagnosis (*p* = 0.012). Overall, these findings suggest that the two groups were broadly comparable in terms of end-of-life clinical status. Notably, although all patients in the palliative group had a documented request for referral, the majority of oncology patients did not receive palliative care consultations (59.5%), with overall consultation rates differing significantly between groups (*p* = 0.020).

**Table 2:** Demographic and clinical characteristics by care group (Oncology vs. palliative).

Variable	Died		Total	*P*-value
	Oncology	Palliative		
Age at death (Group)				
Above or equal 57.9	71 (48.0%)	79 (52.3%)	150 (50.2%)	0.452
Less than 57.9	77 (52.0%)	72 (47.7%)	149 (49.8%)	
Gender				
Female	67 (45.3%)	82 (54.3%)	149 (49.8%)	0.118
Male	81 (54.7%)	69 (45.7%)	150 (50.2%)	
Cancer type				
Breast cancer	25 (16.9%)	29 (19.2%)		**0.031**
Central nervous system	4 (2.7%)	16 (10.6%)	20 (6.7%)	
Gastrointestinal	46 (31.1%)	40 (26.5%)	86 (28.8%)	
Genitourinary	28 (18.9%)	18 (11.9%)	46 (15.4%)	
Gynecological	4 (2.7%)	10 (6.6%)	14 (4.7%)	
Head & neck	7 (4.7%)	4 (2.6%)	11 (3.7%)	
Hematological	2 (1.4%)	6 (4.0%)	8 (2.7%)	
Lung cancer	20 (13.5%)	15 (9.9%)	35 (11.7%)	
Others	3 (2.0%)	7 (4.6%)	10 (3.3%)	
Sarcoma	9 (6.1%)	6 (4.0%)	15 (5.0%)	
Stage at diagnosis				
I	14 (9.5%)	32 (21.2%)	46 (15.4%)	**0.012**
II	21 (14.2%)	22 (14.6%)	43 (14.4%)	
III	29 (19.6%)	35 (23.2%)	64 (21.4%)	
IV	84 (56.8%)	62 (41.1%)	146 (48.8%)	
Last admission stage				
I	1 (0.7%)	3 (2.0%)	4 (1.3%)	0.434
II		1 (0.7%)	1 (0.3%)	
III	7 (4.7%)	4 (2.6%)	11 (3.7%)	
IV	140 (94.6%)	143 (94.7%)	283 (94.6%)	
Reason admission pain				
False	118 (79.7%)	125 (82.8%)	243 (81.3%)	0.499
True	30 (20.3%)	26 (17.2%)	56 (18.7%)	
Fever				
False	137 (92.6%)	142 (94.0%)	279 (93.3%)	0.610
True	11 (7.4%)	9 (6.0%)	20 (6.7%)	
Decrease level of consciousness				
False	122 (82.4%)	121 (80.1%)	243 (81.3%)	0.610
True	26 (17.6%)	30 (19.9%)	56 (18.7%)	
Decrease oral intake				
False	144 (97.3%)	143 (94.7%)	287 (96.0%)	0.253
True	4 (2.7%)	8 (5.3%)	12 (4.0%)	
Bleeding				
False	142 (95.9%)	146 (96.7%)	288 (96.3%)	0.733
True	6 (4.1%)	5 (3.3%)	11 (3.7%)	
Procedure				
False	145 (98.0%)	143 (94.7%)	288 (96.3%)	0.218
True	3 (2.0%)	8 (5.3%)	11 (3.7%)	
Seizure				
False	144 (97.3%)	147 (97.4%)	291 (97.3%)	0.999
True	4 (2.7%)	4 (2.6%)	8 (2.7%)	
Others
False	147 (99.3%)	151 (100%)	298 (99.7%)	0.495
True	1 (0.7%)		1 (0.3%)	
Palliative referral				
No	105 (70.9%)		105 (35.1%)	**<** **0.001**
Yes	43 (29.1%)	151 (100%)	194 (64.9%)	
Palliative Consultation				
No	88 (59.5%)	109 (72.2%)	197 (65.9%)	**0.020**
Yes	60 (40.5%)	42 (27.8%)	102 (34.1%)	

Bold font in *p*-value column reflects statistically significant (*p*-value ≤ 0.05).

### 3.3 Total cost and frequency

As shown in Table 3, a total of 1,623 exams were performed among 299 decedents during the final seven days of life, with an aggregate cost of 186,572.64 USD, comprising 145,870.57 USD for laboratory testing and 40,702.07 USD for radiologic imaging. Patients underwent a mean of 5.43 ± 3.02 exams, with a median of 5 (range 0–12). Laboratory exams were performed a mean of 4.44 ± 2.03 times per patient (median 5, range 1–7), while radiologic exams were performed a mean of 1.91 ± 1.02 times (median 2, range 1–5).

**Table 3:** Total cost and frequency of laboratory and radiology exams during the last 7 days of life: oncology and palliative patients (*N* = 299).

Variable	*N*	Sum	Mean	SD	Median	Min	Max
Days before death	299	498.00	1.67	5.05	1.00	0.00	76.00
Days pal death	194	-	29.69	50.03	10.50	0.00	329.00
Laboratory frequency	277	1,230.00	4.44	2.03	5.00	1.00	7.00
Radiology frequency	206	393.00	1.91	1.02	2.00	1.00	5.00
Total frequency (laboratory & radiology)	299	1,623.00	5.43	3.02	5.00	0.00	12.00
Radiology cost (USD)	206	40,702.07	198.62	246.90	108.93	21.15	1,684.99
Laboratory cost (USD)	277	145,870.57	526.79	319.84	489.29	5.64	1,432.56
Total cost (laboratory & radiology) (USD)	299	186,572.64	625.41	466.45	559.77	0.00	2,442.67

Days before death: Number of days between the last laboratory or radiology exam and the date of death.

Laboratory frequency: Number of laboratory exams for all patients who underwent laboratory testing.

Radiology frequency: Number of radiology exams for all patients who underwent radiology testing.

The mean laboratory cost per patient was 526.79 ± 319.84 USD (median 489.29 USD, range 5.64–1,432.56), and the mean radiology cost was 198.62 ± 246.90 USD (median 108.93 USD, range 21.15–1,684.99). The combined mean exam cost was 625.41 ± 466.45 USD, with a median of 559.77 USD (range 0–2,442.67). The interval between the final exam and death was short, with a median of 1 day (mean 1.67 ± 5.05, range 0–76). Among patients for whom a palliative care referral was requested (*n* = 194), the mean referral-to-death interval was 29.69 ± 50.03 days, with a median of 10.5 days (range 0–329).

### 3.4 Palliative vs. oncology

Healthcare utilization differed significantly between groups (Table 4). Oncology-managed patients incurred significantly higher diagnostic utilization and costs in the final seven days of life compared with palliative care patients. Mean exam costs were more than double in the oncology group (856.58 ± 445.49 USD vs. 397.38 ± 364.39 USD), with corresponding differences in median costs (819.17 USD vs. 302.94 USD; *p* < 0.001). The frequency of laboratory and radiological exams was also higher among oncology patients, with greater mean (6.60 vs. 4.20) and median values (7.00 vs. 4.00; *p* < 0.001), indicating increased diagnostic intensity at end of life.

**Table 4:** Cost and frequency of laboratory and radiology exams and palliative referrals before death for palliative vs. oncology patients.

Variable	Died	*N*	Mean (95% CI)	SD	Median (Min, Max)	*P*-value
Age at death	Oncology	148	57.30 (55.40, 59.10)	13.50	57.10 (20.41, 86.73)	0.899
	Palliative	151	57.00 (55.00, 59.00)	15.00	59.10 (19.29, 84.19)	
Days before death	Oncology	148	0.39 (0.30, 0.47)	0.61	0.00 (0.00, 4.00)	< 0.001
	Palliative	151	2.92 (2.00, 3.84)	6.86	1.00 (0.00, 76.00)	
Days pal ref death	Oncology	43	5.95 (2.29, 9.61)	14.30	1.00 (0.00, 70.00)	< 0.001
	Palliative	151	36.50 (29.10, 43.80)	54.40	14.00 (1.00, 329.00)	
Laboratory cost (USD)	Oncology	147	648.42 (598.87, 696.95)	307.44	630.27 (21.15, 1432.56)	< 0.001
	Palliative	130	388.30 (340.82, 436.05)	275.46	338.40 (5.64, 1188.63)	
Laboratory Frequency	Oncology	147	4.88 (4.61, 5.16)	2.01	6.00 (1.00, 7.00)	< 0.001
	Palliative	130	3.94 (3.66, 4.22)	1.93	4.00 (1.00, 7.00)	
Radiology cost (USD)	Oncology	128	245.34 (204.45, 286.23)	198.00	153.69 (20.85, 1681.29)	< 0.001
	Palliative	78	121.12 (92.14, 150.87)	155.10	68.39 (21.94, 1081.17)	
Radiology frequency	Oncology	128	2.07 (1.91, 2.23)	1.11	2.00 (1.00, 5.00)	0.008
	Palliative	78	1.64 (1.49, 1.79)	0.79	1.00 (1.00, 5.00)	
Total cost (laboratory & radiology) (USD)	Oncology	148	856.58 (784.35, 927.50)	445.49	819.17 (0.00, 2443.36)	< 0.001
	Palliative	151	397.38 (339.45, 454.35)	364.39	302.94 (0.00, 1650.65)	
Total frequency (laboratory & radiology)	Oncology	148	6.60 (6.20, 7.10)	2.66	7.00 (0.00, 12.00)	< 0.001
	Palliative	151	4.20 (3.80, 4.70)	2.89	4.00 (0.00, 12.00)	

Days before death: Number of days between the last laboratory or radiology exam and the date of death.

Laboratory frequency: Number of laboratory exams for all patients who underwent laboratory testing.

Radiology frequency: Number of radiology exams for all patients who underwent radiology testing.

Laboratory costs were significantly higher among oncology patients (mean 648.42 ± 307.44 USD; median 630.27 USD) than palliative patients (mean 388.30 ± 275.46 USD; median 338.40 USD; *p* < 0.001), accompanied by a higher frequency of laboratory testing (mean 4.88 ± 2.01 vs. 3.94 ± 1.93; median 6.00 vs. 4.00; *p* < 0.001). Similarly, oncology patients had higher radiologic costs (mean 245.34 ± 198.00 USD vs. 121.12 ± 155.10 USD; median 153.69 USD vs. 68.39 USD; *p* < 0.001) and underwent imaging more frequently (mean 2.07 ± 1.11 vs. 1.64 ± 0.79; median 2 vs. 1; *p* = 0.008).

The timing of exams also differed significantly. The median number of days between the final exam and death was 0 days for oncology patients compared with 1 day for palliative patients (*p* < 0.001). Additionally, patients who died under palliative care had a significantly longer interval from palliative care referral request to death compared to oncology-managed patients, including those with a referral request who were not subsequently seen by the palliative care service (mean 36.50 ± 54.40 vs. 5.95 ± 14.30 days; median 14.00 vs. 1.00 day; *p* < 0.001).

**Figure 1:**
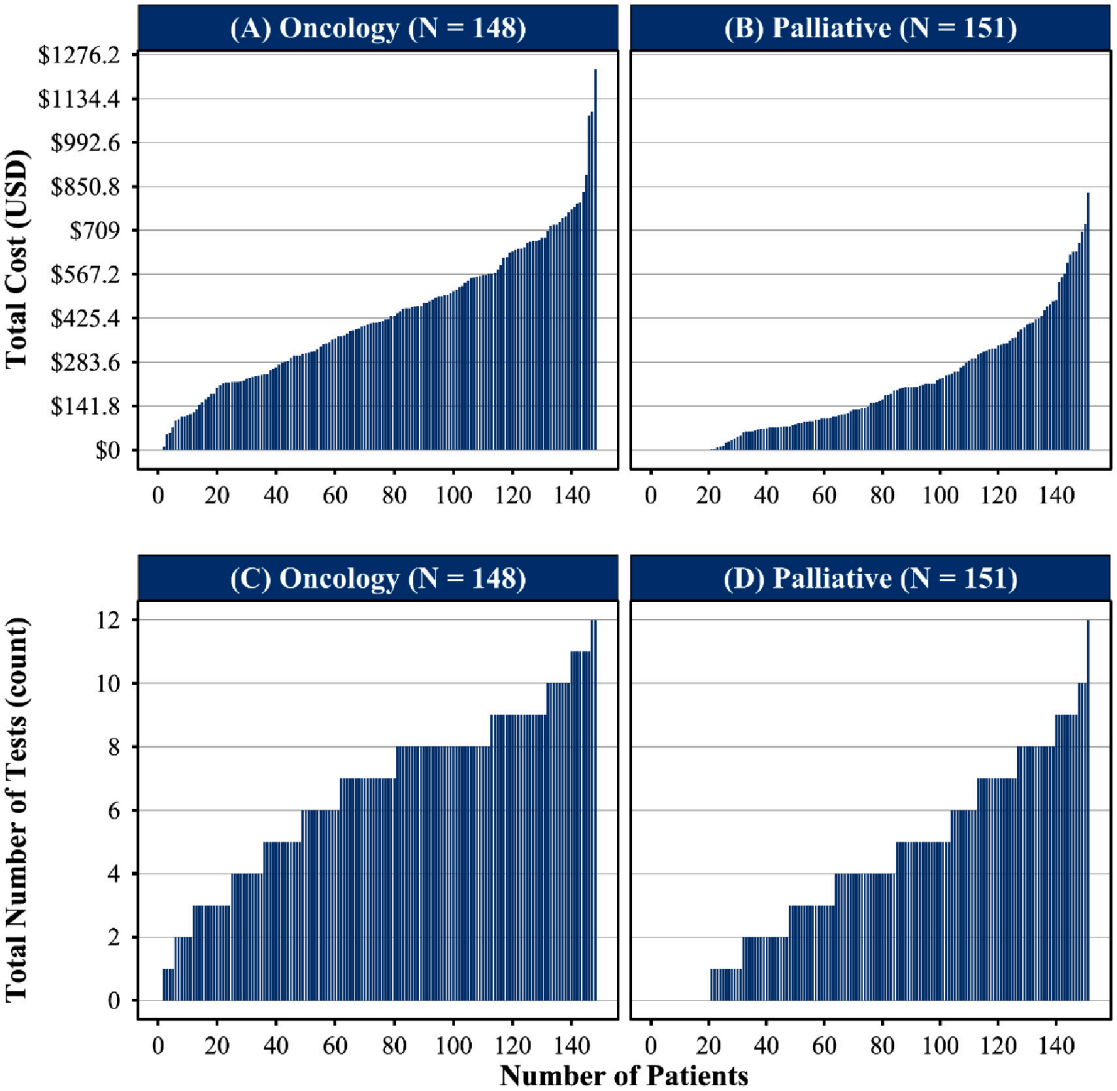
Distribution of total healthcare costs and diagnostic utilization among deceased cancer patients by care model. **(A, B)** Display total cost per patient (USD), sorted in ascending order, for patients managed under oncology care (*N* = 148) and palliative care (*N* = 151), respectively. **(C, D)** Illustrate the frequency of laboratory and imaging exams per patient in the same groups. Each bar represents an individual patient. Patients receiving palliative care demonstrate a lower and more gradual increase in total costs and diagnostic utilization compared to those under oncology care.

Among oncology patients, only one individual did not undergo any laboratory testing during the final week of life, compared with 21 patients in the palliative cohort. Likewise, 20 oncology patients had no radiologic exams, whereas 73 palliative patients received no imaging. Figure 1 illustrates the distribution of total exam costs by service at time of death, demonstrating both higher expenditure levels and greater variability among oncology-managed patients relative to those receiving palliative care.

## 4 Discussion

This study highlights pronounced disparities in end-of-life healthcare utilization and associated costs between oncology patients receiving standard oncologic care and those receiving palliative care services. In the present study, baseline characteristics were largely comparable between oncology and palliative care groups, particularly in terms of age, gender, reasons for admission, and advanced disease status at the end of life. The predominance of stage IV disease in both groups suggests a similar illness trajectory, reducing the likelihood that differences in outcomes were driven solely by disease severity. Although variations in cancer type and stage at diagnosis were observed, these are unlikely to fully account for the substantial differences in healthcare utilization. Consistent with our findings, prior studies have shown that integration of palliative care is associated with reduced use of intensive interventions and lower healthcare costs near the end of life, independent of baseline characteristics (3, 16). More recent evidence further supports these observations, demonstrating that early palliative care involvement leads to less aggressive care, fewer hospital-based interventions, and improved resource utilization without compromising survival (4). Together, these findings reinforce that the observed differences are more likely attributable to variations in care approach rather than underlying patient characteristics. Patients receiving palliative care exhibited markedly lower frequencies and costs of laboratory and radiologic exams, aligning with prior evidence that early integration of palliative care mitigates non-beneficial and burdensome interventions near the end of life (3). In our study, patients under palliative care had a longer interval from referral request to death than oncology-managed patients, with a median interval of 14 days. While prior oncology admissions may contribute, the majority of oncology patients with a palliative care referral request who were not subsequently seen had much shorter intervals, suggesting that the longer duration among palliative care patients primarily reflects the timing and extent of palliative care involvement. This pattern aligns with the global trend of late palliative engagement despite recommendations for earlier integration (17).

Notably, several patients in our study requested palliative care referral yet continued to receive care solely under oncology services and died without palliative involvement. This gap may be related to delayed referrals by oncology teams, patient or family reluctance, or the insufficient coordination between oncology and palliative care services. This pattern reflects a common perception in many countries that palliative care is primarily end-of-life care, as highlighted in a study from Brazil by Cruz et al. (18), which reported that the majority of cancer patients were referred very late to palliative care services, with 85.3% receiving less than 2 months of follow-up and most dying in the hospital. Our findings are consistent with prior evidence showing substantial underutilization of palliative care before death; for instance, Hui et al. reported that only 27% of patients with advanced thoracic malignancies received outpatient palliative care prior to death. Such delayed engagement limits opportunities for symptom control, advance care planning, and avoidance of unnecessary acute care (19). Further research is needed to clarify patient, family, and system-level barriers to timely palliative integration.

Decreased level of consciousness and pain crisis were the most frequent indications for the final hospitalization, reflecting the high symptom burden experienced by patients with advanced cancer near the end of life. This finding is consistent with previous studies showing that uncontrolled pain and acute neurological deterioration are among the leading drivers of terminal hospital admissions in oncology populations. Prior research has reported similar patterns, with pain crises and altered mental status accounting for a substantial proportion of admissions to acute palliative and emergency care services in the last weeks of life (20). These observations reinforce evidence that delayed or inadequate symptom control may contribute to potentially avoidable hospitalizations and underscore the importance of earlier palliative care integration to optimize symptom management and reduce end-of-life acute care utilization (21).

The majority of patients in this cohort presented with advanced-stage malignancies, particularly gastrointestinal and breast cancers, conditions well-recognized for high symptom burden and complex end-of-life trajectories. Lower diagnostic intensity among palliative patients in our study reinforce accumulating evidence that reduced diagnostic intensity does not compromise, and may even enhance, care quality when care is aligned with patient-centered goals and symptom-directed management (22). Similarly, the landmark randomized controlled trial by Temel et al. (3) demonstrated that early palliative care in metastatic non-small-cell lung cancer not only reduced aggressive and costly interventions at the end of life but also improved quality of life and, notably, extended survival, emphasizing that high-value care and judicious resource utilization are compatible.

Economic analyses further underscore the impact of palliative integration. In our cohort, palliative care recipients incurred more than a 50% reduction in mean total costs during the end-of-life period. In resource-constrained healthcare systems, such cost savings are particularly consequential and provide a compelling argument for broader implementation of structured palliative referral pathways, standardized needs-based triggers, and enhanced multidisciplinary collaboration. Collectively, these findings support institutional and national strategies that prioritize timely palliative integration to enhance patient-centered outcomes while containing escalating healthcare expenditures.

### 4.1 Strengths and limitations

This study's strengths include its use of comprehensive electronic medical records from a leading cancer center, capturing real-world clinical practice in patients with advanced-stage cancers. Direct comparison between oncology and palliative care pathways provides insights into diagnostic utilization and costs, while economic analysis underscores the added value of palliative integration. Limitations include the retrospective, single-center design, which limits causal inference and generalizability, and introduces potential residual confounding and selection bias. Additionally, the short median interval of 14 days from referral to death limits the ability to assess the impact of earlier palliative care integration. Nevertheless, despite some referrals occurring very late in the disease trajectory, patients managed by palliative care still demonstrated lower diagnostic intensity and associated costs, suggesting a measurable impact even with late integration. Furthermore, reasons for late referral such as patient and family preferences were not systematically captured, and reliance on records may introduce misclassification. We also acknowledge that detailed data on chemotherapy administration near the end of life, an important quality indicator, were not collected; this represents an important area for future research.

## 5 Conclusion

Integration of palliative care within oncology practice at KHCC was associated with substantially lower diagnostic utilization and costs during the final days of life. Earlier referral to palliative services has the potential to optimize symptom management, align care with patient goals, and reduce healthcare expenditures. Future multicenter and prospective studies should investigate patient-centered outcomes and systematically evaluate barriers to timely palliative engagement to inform policies that promote high-value, patient-centered end-of-life care.

## Data Availability

The original contributions presented in the study are included in the article/supplementary material, further inquiries can be directed to the corresponding authors.
